# Changing Epidemiology of Hepatitis A and Hepatitis E Viruses in China, 1990–2014

**DOI:** 10.3201/eid2302.161095

**Published:** 2017-02

**Authors:** Xiang Ren, Peng Wu, Liping Wang, Mengjie Geng, Lingjia Zeng, Jun Zhang, Ningshao Xia, Shengjie Lai, Harry R. Dalton, Benjamin J. Cowling, Hongjie Yu

**Affiliations:** Key Laboratory of Surveillance and Early-Warning on Infectious Disease, Chinese Center for Disease Control and Prevention, Beijing, China (X. Ren, L. Wang, M. Geng, L. Zeng, S. Lai, H. Yu)**;**; World Health Organization Collaborating Centre for Infectious Disease Epidemiology and Control, Li Ka Shing Faculty of Medicine, The University of Hong Kong, Hong Kong, China (X. Ren, P. Wu, B. Cowling)**;**; State Key Laboratory of Molecular Vaccinology and Molecular Diagnostics, National Institute of Diagnostics and Vaccine Development in Infectious Diseases, Xiamen University School of Public Health, Xiamen, China (J. Zhang, N. Xia);; University of Southampton, Southampton, UK (S. Lai);; Royal Cornwall Hospital and European Centre for Environment and Human Health, University of Exeter, Truro, UK (H.R. Dalton);; Fudan University School of Public Health, Key Laboratory of Public Health Safety, Ministry of Education, Shanghai, China (H. Yu)

**Keywords:** hepatitis A, hepatitis E, epidemiology, China, viruses, vaccine, vaccination

## Abstract

We compared the epidemiology of hepatitis A and hepatitis E cases in China from 1990–2014 to better inform policy and prevention efforts. The incidence of hepatitis A cases declined dramatically, while hepatitis E incidence increased. During 2004–2014, hepatitis E mortality rates surpassed those of hepatitis A.

Hepatitis A virus (HAV) and hepatitis E virus (HEV) cause acute hepatitis in humans and are transmitted mainly through the fecal–oral route. Hepatitis A and hepatitis E became notifiable in China in 1990 and 1996, respectively. Since the introduction of the hepatitis A vaccine and the start of mass vaccination in several countries in the 1980s, hepatitis A incidence declined substantially, not only among vaccinated children but in the population as whole (*1*,*2*). China first licensed its live attenuated hepatitis A vaccine in 1992 and later the inactivated hepatitis A vaccine in 2002 (*3*). The hepatitis A vaccine was initially introduced into the private market, although some provinces provided subsidies through the World Health Organization Expanded Programme on Immunization (http://www.wpro.who.int/china/areas/immunization/en/). Starting in May 2008, hepatitis A vaccinations were incorporated into the routine national childhood immunization program for children >18 months of age (*3*).

HEV is a substantial cause of illness and death worldwide, particularly among pregnant women (*4*). Until the introduction of the first hepatitis E vaccine to private markets in China in 2011, there were no specific pharmaceutical interventions for HEV (*5*). Given the similarity in diseases caused by HAV and HEV and the recent decline in hepatitis A incidence, we compared the epidemiology of human cases infected with the 2 pathogens in China.

## The Study

We obtained data on cases of hepatitis A reported during 1990–2014 and hepatitis E for 1997–2014 from China’s National Notifiable Disease Report System and collated demographic information from the China National Bureau of Statistics. We defined confirmed cases on the basis of dates of disease onset and updated diagnostic criteria issued by the Chinese Ministry of Health in 2008; these critera are based on epidemiologic history, clinical signs, and laboratory test results (Technical Appendix Table)

We used R version 3.0.1 (R Foundation for Statistical Computing, Vienna, Austria) and SAS version 9.2 (SAS Institute Inc., Cary, NC, USA) to estimate annual incidence and mortality rates for hepatitis A and hepatitis E according to patient age and sex. Notified cases were geocoded into provinces and mapped by ArcGIS 10 (Esri Inc., Redlands, CA, USA). To examine seasonality, we created heat maps by using monthly incidence normalized to the maximum incidence each year. We used a similar approach to examine seasonality across latitudes. We assessed potential associations between incidence and demographic and economic factors by using Poisson regression.

**Figure 1:**
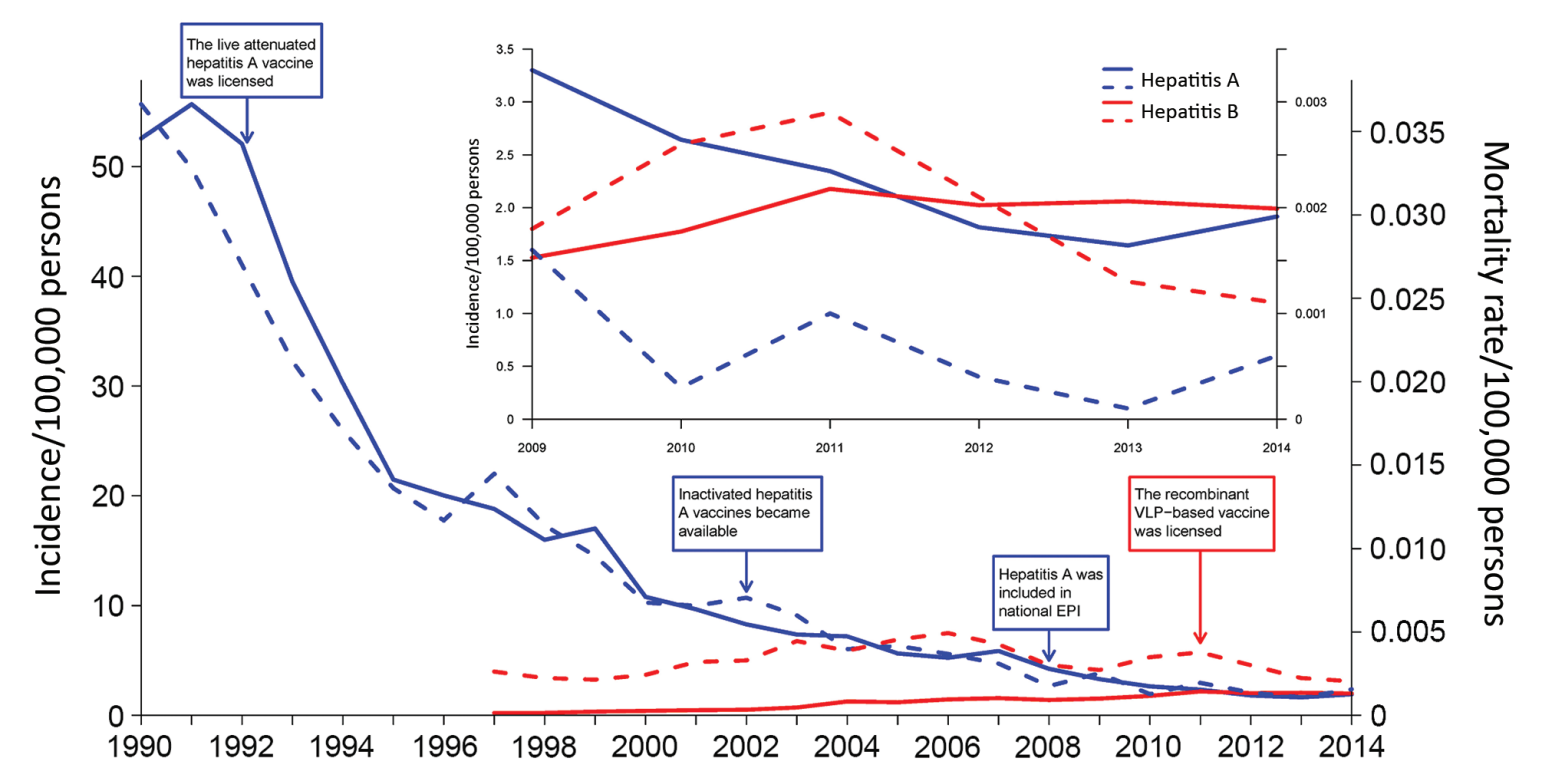
Annual incidence (solid lines) and mortality rates (dashed lines) of notified hepatitis A (blue) and E (red) cases in China, 1990-2014. The inset shows an enlarged view of rates during 2009–2014. EPI, Expanded Program on Immunization; VCP, virus-like particle.

Hepatitis A incidence dropped from 55.7 cases/100,000 person-years in 1991 to 1.9 cases/100,000 person-years in 2014, a decrease of 96.6% (Figure 1). In contrast, hepatitis E incidence increased significantly over this period, from 0.21 cases/100,000 person-years in 1997 to 1.99 cases/100,000 person-years in 2014, an 8-fold increase (p<0.0001 by Poisson regression) (Figure 1). The mortality and incidence rates for hepatitis E overtook those for hepatitis A in 2004 and 2011, respectively (Figure 1). Hepatitis E cases across the country were most frequently reported in March (Technical Appendix Figures 4, 5). This change may result from increased temperature and rainfall in the spring, which could increase the likelihood of acquiring HEV infection from exposure to contaminated water, such as water sourced from a stream near a free-range pig farm (*6*–*8*). In contrast, the seasonal pattern of HAV infections varied by latitude; cases were reported most frequently in the spring in southern provinces and in the autumn in most northern provinces (Technical Appendix Figure 5). The decline in the incidence of hepatitis A cases in winter and summer varies by province, and reasons for the differential latitudinal pattern are unclear (*7**–**9*).

**Figure 2:**
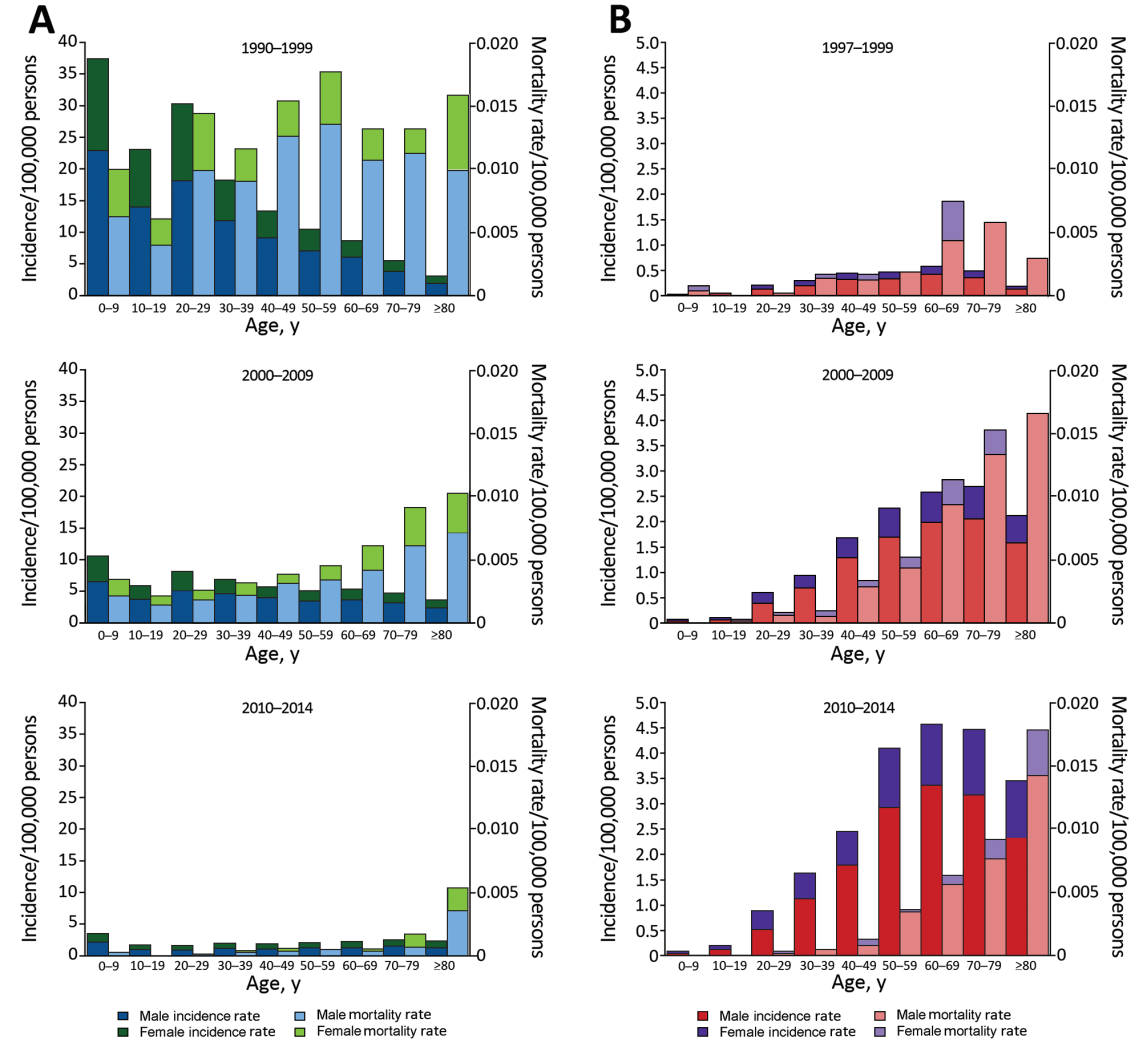
Age distribution of patients with reported cases of A) hepatitis A (blue, male patients; green, female patients) and B) hepatitis E (red, male patients; purple, female patients) in China for 1990–1999, 2000–2009, and 2010–2014. Incidence and mortality rates were calculated in 10-year age groups for the 3 periods. For each color, the darker shade represents incidence and the lighter shade represents mortality rate.

Hepatitis A incidence was highest among children and young adults (Figure 2). In contrast, hepatitis E incidence was highest among older adults and low among children and young adults (Figure 2). For both diseases, incidence and mortality rates were higher among male patients, and mortality rates tended to increase with age (Figure 2). The percentage of infections resulting in death were generally similar among men and women within each age group for most years (Technical Appendix Figure 2).

We found considerable changes in the epidemiology of hepatitis A and E over time in mainland China. The major decline in hepatitis A incidence during 1992–2014 cannot be explained solely by the introduction of the vaccine because implementation of vaccinations in the general population had been relatively low until the inactivated hepatitis A vaccine was included in the national Expanded Program on Immunization in May 2008 (*3*). Other key reasons could be heightened public awareness, improved social hygiene, and upgrades in sewage treatment and water quality (*9*–*11*). Decreasing incidence was also accompanied by a change in the age distribution of reported hepatitis A case-patients; the average age increased over time (Figure 2), possibly a consequence of inclusion of the vaccine in the national Expanded Program on Immunization, which targets children >18 months of age. The incidence of hepatitis A is now highest in northwestern China, which is a comparatively less developed region of the country (Technical Appendix Figure 1).

Our findings in China are similar to those documented in other countries, where HEV infection is now more common than HAV infection (*12*,*13*). The increase in hepatitis E incidence could result from either a true increase in the number of cases or from improved case diagnosis. Hepatitis E case-patients are mostly adults, particularly older adults (Figure 2). Although we did not have data on HEV genotypes for this study, it is possible that the change in age distribution of hepatitis E patients may result from the shift of the prevalent HEV genotype in China from genotype 1 to genotype 4 (and, to a lesser extent, genotype 3). Genotype 3 and genotype 4 are known to infect older men (*14*,*15*). The increase in the number of HEV infections in eastern China (Technical Appendix Figure 1) could also be caused by this genotype shift and improved surveillance in these more developed provinces (*15*), rather than by true geographic heterogeneity in risk factors. 

There are some limitations to our study (Technical Appendix). Our findings are inferred from case notification data, and the data quality could vary because of changes in case definitions. Other limitations include variable availability of laboratory diagnostics and lack of hepatitis E genotype data. 

## Conclusions

Reports of hepatitis A in China have declined substantially, while reports of hepatitis E cases have continued to rise. The mortality rate for hepatitis E surpassed that for hepatitis A in 2004. Decreasing trends of hepatitis A incidence after implementation of a vaccination program targeting children >18 months of age indicate a similar strategy for hepatitis E could be considered as a means to curtail incidence. In addition, variations in demographic, geographic and seasonal distributions of hepatitis A and E may inform future prevention strategies in China.

Technical Appendix Results summary, diagnostic criteria used for hepatitis A and hepatitis E infections, and spatial and temporal distribution and patterns of hepatitis A and hepatitis E viruses, China.
