# Correction: Vol. 23, No. 2

**DOI:** 10.3201/eid2303.C22303

**Published:** 2017-03

**Keywords:** errata, erratum, correction

The key in the Figure 1 inset should have referred to hepatitis A and E in Changing Epidemiology of Hepatitis A and Hepatitis E Viruses in China, 1990–2014 (X. Ren et al.). The article has been corrected online (http://wwwnc.cdc.gov/eid/article/23/2/16-1095_article).

