# Supplementary material for: Changing Epidemiology of Hepatitis A and Hepatitis E Viruses in China, 1990–2014
Source: Emerg Infect Dis. 2017 Feb;23(2):276–9. doi: 10.3201/eid2302.161095 (PMC5324787; doi:10.3201/eid2302.161095)
Supplement: Technical Appendix — Results summary, diagnostic criteria used for hepatitis A and hepatitis E infections, and spatial and temporal distribution and patterns of hepatitis A and hepatitis E viruses, China. [file 16-1095-Techapp-s1.pdf]

Article DOI: http://dx.doi.org/10.3201/eid2302.161095

# Changing Epidemiology of Hepatitis A and Hepatitis E Viruses in China, 1990–2014

## Technical Appendix

## Regional Hepatitis Incidence

Provinces along the Yangtze River and some northeastern provinces had relatively higher hepatitis A incidence in the 1990s, while a higher incidence has been observed in western China since 2000 (Technical Appendix Figure 1). In contrast, hepatitis E incidence s have remained highest in eastern China (Technical Appendix Figure 1). In the Poisson regression models, both GDP and GDP per capita (Technical Appendix Figure 3) showed a strong association with the incidence s of hepatitis A ($p < 0.0001$) and the incidence s of hepatitis E ($p < 0.0001$). GDP and GDP per capita were inversely correlated with incidence of hepatitis A but positively associated with the incidence of hepatitis E.

## Study Limitations

There are some potential limitations to our work. First, the data quality may be influenced by changes in the national notifiable disease reporting system including changes in case definitions, reporting methods, availability of health facilities and laboratory diagnostics, under reporting, and completeness and accuracy of data over the years. Diagnosis and notification of cases may vary across the country, affecting our geographic comparisons of incidence. Second, we inferred the relative disease burden of hepatitis A and E on the basis of case notification data, but underestimation of hepatitis A or E cases may have biased our comparison since many cases may not have been diagnosed and reported. Information on hepatitis E genotypes was not available in the case reports, and therefore we could not explore the potential effect of changing patterns in the genotypes on HEV infections in. In addition, the increase in hepatitis E could be due to increased awareness of the disease and use of diagnostic testing and. We cannot be certain if there has been a true increase in incidence as data on the total number of cases tested for HEV

over the study period are not available. Finally, we did not have information on the total number of laboratory tests performed over time, and increase in laboratory capacity may have led to gradual increases in the incidence of notified cases of hepatitis A and E over time.

**Technical Appendix Table.** Change of diagnostic criteria for viral hepatitis A and viral hepatitis E in China*

| Standard Name | Diagnostic Criteria and Principles of Management for Viral Hepatitis A | Diagnostic Criteria of Viral Hepatitis A | Diagnostic Criteria and Principles of Management for Viral Hepatitis E | Diagnostic Criteria for Hepatitis E |
|---|---|---|---|---|
| Chinese Standard Number | GB 17010—1997 | WS 298–2008 | GB 17011—1997 | WS 301–2008 |
| Issued by | Chinese Ministry of Health | Chinese Ministry of Health | Chinese Ministry of Health | Chinese Ministry of Health |
| Date issued | 1997/10/6 | 2008/12/11 | 1997/10/6 | 2008/12/11 |
| Date Enforced | 1998/10/1 | 2009/6/15 | 1998/10/1 | 2009/6/15 |
| Epidemiologic Linkage | 1.1 has epidemiologic history of contact with acute hepatitis A patient or having ingested contaminated food or water in 45 d before onset of illness | 1.1 has epidemiologic history of contact with a laboratory-confirmed acute hepatitis A patient or has ingested contaminated food or water in 2–7weeks before onset of illness, or lives in the place with recent hepatitis A epidemic or outbreak, or has history of travel to hepatitis A epidemic area | 1.1 has epidemiologic history of contact with hepatitis patient or ingested contaminated food or water in 2–6 weeks before onset of illness or has history of travel to high hepatitis E prevalence area or hepatitis E epidemic area | 1.1 has epidemiologic history of contact with a laboratory-confirmed hepatitis E patient, or ingested contaminated food or water in 15–75 d before onset of illness, or has history of travel to hepatitis E high prevalence area or hepatitis E epidemic area |
| Clinical Description | 2.1 has symptoms of fever, fatigue, loss of appetite, nausea and vomiting in the recent week with exclusion of other diseases, and has hepatomegaly with tenderness or percussion pain<br>2.2 has jaundice with exclusion of other diseases<br>2.3 has Obstructive jaundice for over 3 weeks with exclusion of other diseases<br>2.4 has acute onset with severe gastrointestinal symptoms and neuropsychiatric symptoms occur within 10 d since the onset(over grade 2 of the Parsons-Smith Scale of Hepatic Encephalopathy) with exclusion of other diseases<br>2.5 has a rapidly shrinking liver<br>2.6 starts with onset of acute hepatitis, has extreme fatigue, severe loss of appetite, rapidly deepened jaundice, ascites and bleeding tendency, progressively shrinking liver. During 10 d to 8 weeks since the onset has impaired consciousness (over grade 2 of the Parsons-Smith Scale of Hepatic Encephalopathy) with exclusion of other diseases<br>2.7 has liver pathological characteristics | 2.1 has symptoms of fever, fatigue, loss of appetite, nausea, vomiting, or abdominal distension, constipation and etc. and has hepatomegaly with tenderness or percussion pain<br>2.2 has jaundice with exclusion of other diseases | 2.1 has symptoms of fatigue, loss of appetite lasting over 1 week or other gastrointestinal symptom or hepatomegaly with tenderness or percussion pain with exclusion of other diseases<br>2.2 has jaundice with exclusion of other diseases<br>2.3 has jaundice rapidly deepened<br>2.4 has mental or neurologic symptoms (Hepatic Encephalopathy) within 10 d after the onset with exclusion of other reasons<br>2.5 has severe abdominal distention or ascites | 2.1 has symptoms of fatigue, loss of appetite, or other gastrointestinal symptom and/or hepatomegaly with tenderness or percussion pain with exclusion of other diseases<br>2.2 has dark urine and jaundice with exclusion of other diseases<br>2.3 for patients with liver failure, has progressive symptoms like fatigue, gastrointestinal symptoms and jaundice, with ascites and/or neuropsychiatric symptoms (dysphoria, disorientation, even delirious, drowsiness and coma) |
| Laboratory Tests | 3.1 has abnormal serum alanine aminotransferase (ALT)<br>3.2 tests positive for the IgM antibody to hepatitis A virus by MAC ELISA or tests IgG antibody to hepatitis A virus 4 times of increasing by competitive inhibition ELISA | 3.1 has increasing serum alanine aminotransferase (ALT)<br>3.2 has total serum bilirubin (TBIL) over 1 times larger than the normal upper limits and/or positive urinary bilirubin tests | 3.1 has increasing serum alanine aminotransferase (ALT)<br>3.2 excludes the acute hepatitis A/B/C/G through serum test<br>3.3 has serum bilirubin(BIL)>17.1 μmol/L (>10mg/L) or positive | 3.1 tests positive for the IgG antibody to hepatitis E virus and/or IgM antibody to hepatitis E virus by EIA kit<br>3.2 has increasing serum alanine aminotransferase (ALT) |

| Standard Name | Diagnostic Criteria and Principles of Management for Viral Hepatitis A | Diagnostic Criteria of Viral Hepatitis A | Diagnostic Criteria and Principles of Management for Viral Hepatitis E | Diagnostic Criteria for Hepatitis E |
|---|---|---|---|---|
| | 3.3 has serum bilirubin (BIL)>17.1 µmol/L and positive urinary bilirubin tests<br>3.4 has serum bilirubin (BIL) increased, especially the direct bilirubin,with alkalinephosphatase(ALP), glutaminepeptideenzyme(GGT) and cholesterol increased, and serum alanine moderately increased<br>3.5 has abnormal liver function, serum bilirubin increased to over 171 µmol/L within a few days or increased by more than 17.1 µmol/L per day, and prothrombin activity less than 40%<br>3.6 has abnormal liver function, transaminase level decreased but the serum bilirubin continued to be elevated, Albumin globulin ratio(A/G)<1, cholesterol decreased and prothrombin activity less than 40% | 3.3 tests positive for the IgM antibody to hepatitis A virus by ELISA or tests IgG antibody to hepatitis A virus 4 times of increasing by competitive inhibition ELISA or tests hepatitis A RNA positive by RT-PCR | urinary bilirubin tests<br>3.4 tests positive for the IgM antibody to hepatitis E virus or tests IgG antibody to hepatitis E virus from negative to positive or titer from low to high or high to low with over quadruple of change by EIA kit<br>3.5 serum bilirubin increased to over 171 µmol/L<br>3.6 has prolonged prothrombin time and prothrombin activity less than 40%<br>3.7 serum bilirubin increased by 17.1 µmol/L daily | 3.3 has total serum bilirubin (TBIL) >17.1 µmol/L (>10mg/L) and/or positive urobilirubin tests<br>3.4 for patients with liver failure, has prothrombin activity progressively decreased to less than 40%<br>3.5 excludes the acute hepatitis A/B/C from serum test |
| Diagnosis and Classification | 4.1 Acute anicteric hepatitis<br>4.1.1 Probable case:2.1+3.1<br>4.1.2 Confirmed case: 4.1.1+3.2<br>4.2 Acute icteric hepatitis<br>4.2.1 Probable case: 2.1+2.2+3.1+3.3<br>4.2.2 Confirmed case: 4.2.1+3.2<br>4.3 Cholestasis hepatitis<br>4.3.1 Probable case: 2.1+2.2+2.3+3.4<br>4.3.2 Confirmed case: 4.3.1+3.2 or 2.7+3.2<br>4.4 Acute severe hepatitis<br>4.4.1 Probable case: 2.4+2.5+3.5<br>4.4.2 Confirmed case: 4.4.1+3.2 or 2.5+2.7<br>4.5 Sub-acute severe hepatitis<br>4.5.1 Probable case: 2.6+3.6<br>4.5.2 Confirmed case:4.5.1+3.2 or 2.7+3.2 | 4.1 Hepatitis A<br>4.1.1 Probable case:1.1+2.1+2.2+3.1 or 1.1+2.1+2.2+3.1+3.2 or 2.1+2.2+3.1 or 2.1+2.2+3.1+3.2<br>4.1.2 Confirmed case: 4.1.1+3.3<br>4.2 Acute anicteric hepatitis A<br>4.2.1 Probable case:1.1+2.1 or 1.1+3.1 or 2.1+3.1<br>4.2.2 Confirmed case: 4.2.1+3.3<br>4.3 Acute icteric hepatitis A<br>4.3.1 Probable case: 1.1+2.1+2.2+3.2 or 1.1+2.2+3.1+3.2 or 2.1+2.2+3.1+3.2<br>4.3.2 Confirmed case: 4.3.1+3.3 | 4.1 Acute anicteric hepatitis E<br>4.1.1 Probable case:2.1+3.1+3.2<br>4.1.2 Confirmed case: 4.1.1+3.4<br>4.2 Acute icteric hepatitis E<br>4.2.1 Probable case: 2.1+2.2+3.1+3.2+3.3<br>4.2.2 Confirmed case: 4.2.1+3.4<br>4.3 Acute severe hepatitis E<br>4.3.1 Probable case: 2.1+2.2+2.3+3.1+3.2+3.3+3.5<br>4.3.2 Confirmed case:4.3.1+2.4+3.6<br>4.4 Sub-acute severe hepatitis E<br>4.4.1 Probable case: 2.1+2.2+2.5+3.1+3.2+3.3+3.5 or 2.1+2.2+2.5+3.1+3.2+3.3+3.7<br>4.4.2 Confirmed case:4.4.1+3.6 or 4.4.1+2.4+3.6 | 4.1 Acute anicteric hepatitis E<br>4.1.1 Probable case:1.1+2.1+3.2+3.5<br>4.1.2 Confirmed case: 4.1.1+3.1<br>4.2 Acute icteric hepatitis E<br>4.2.1 Probable case: 1.1+2.1+2.2+3.2+3.3+3.5<br>4.2.2 Confirmed case: 4.2.1+3.1<br>4.3 Hepatitis E with acute liver failure<br>4.3.1 Probable case: 1.1+2.1+2.2+3.2+3.3+3.5+2.3a+ 3.4a<br>a: has these symptoms within 14 d since the onset<br>4.3.2 Confirmed case: 4.3.1+3.1<br>4.4 Hepatitis E with sub-acute liver failure<br>4.4.1 Probable case: 1.1+2.1+2.2+3.2+3.3+3.5+2.3b+ 3.4b<br>b: has these symptoms after 14 d till 6 mo since the onset<br>4.4.2 Confirmed case:4.4.1+3.1 |

*The revised diagnostic criteria in 2008 provided a simplified disease categorization for hepatitis A and clarified laboratory confirmation for IgG and IgM antibody using ELISA and HAV RNA using RT-PCR. The hepatitis E laboratory testing guidelines were changed in 2008 to include quantification of IgG and IgM antibody using suggested assays.

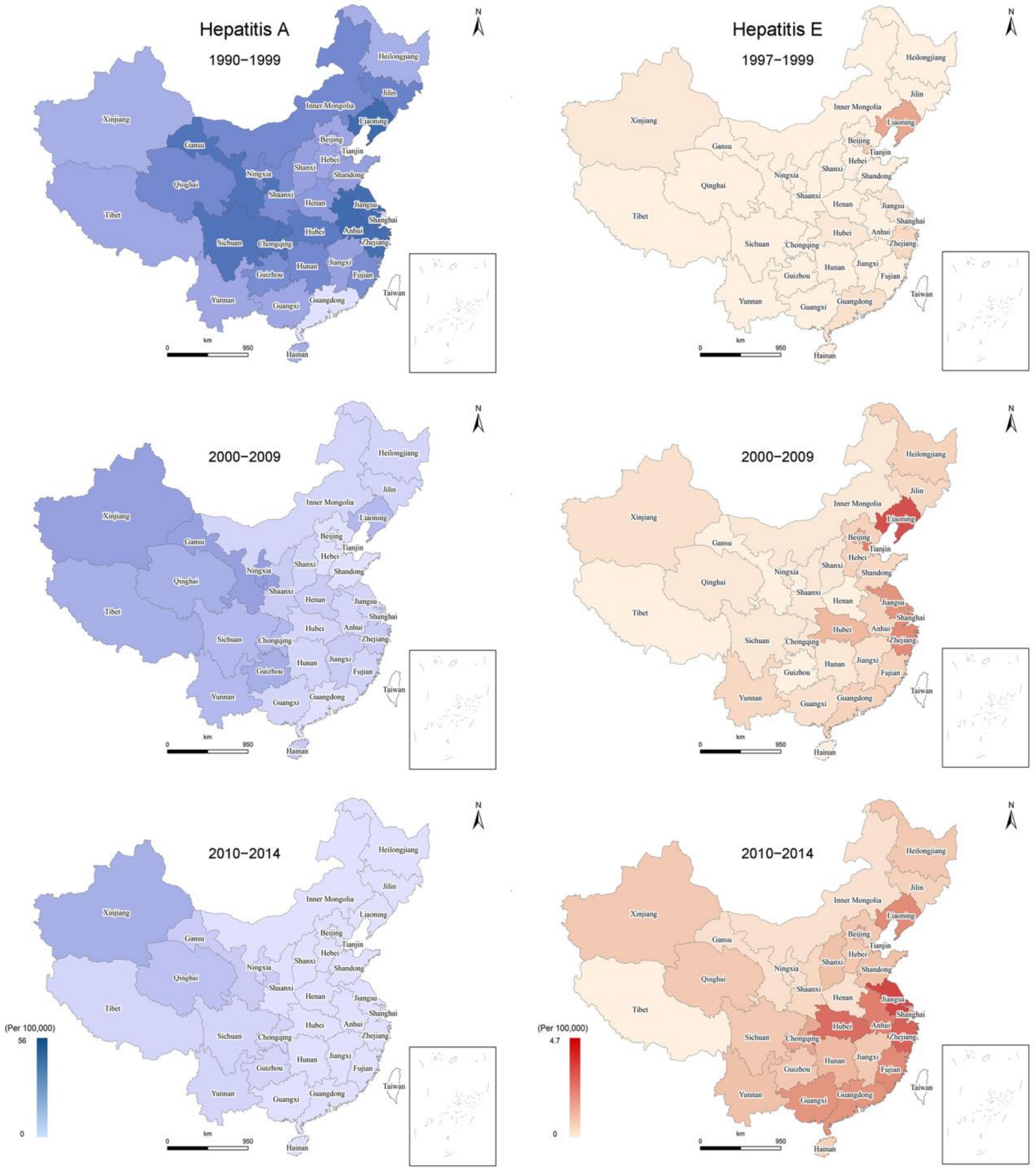


**Technical Appendix Figure 1.** Averaged annual incidence of notifications of hepatitis A (left column) and hepatitis E (right column) in each province of China in 1990–1999, 2000–2009 and 2010–2014. Data were not available on hepatitis A and E cases in Hong Kong SAR, Macau SAR, and Taiwan. Chongqing municipality has been administratively separated from Sichuan province since 1997, and therefore estimates of incidence and mortality rates of hepatitis A in Sichuan province before 1997 were calculated by using data that included Chongqing.

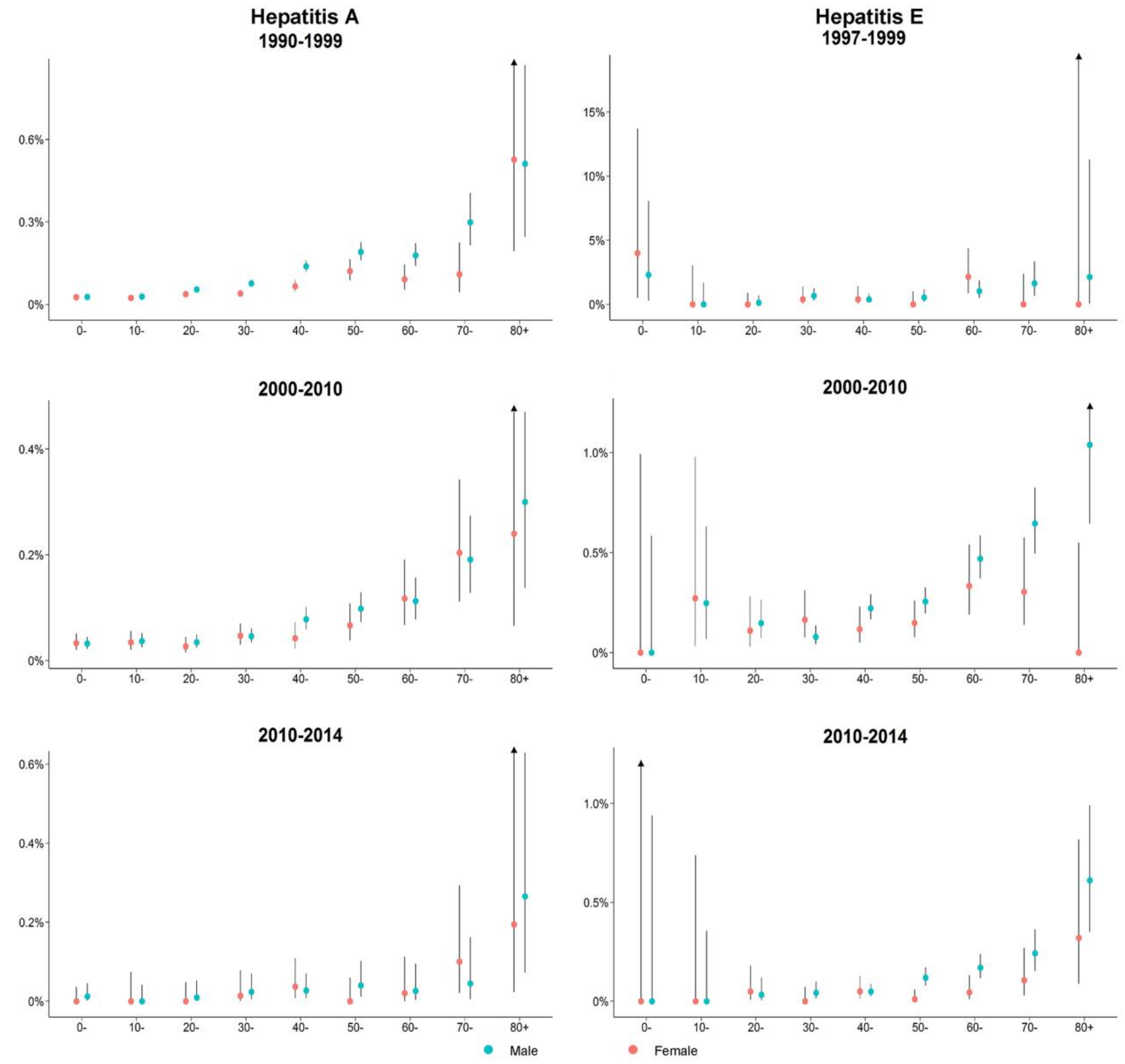


**Technical Appendix Figure 2.** Point estimates (dots) and 95% confidence intervals (vertical gray lines) of the case fatality ratio (defined as notified deaths divided by all notifications) of hepatitis A and E by age group among males (blue) and females (red) in 1990–1999, 2000–2009 and 2010–2014.

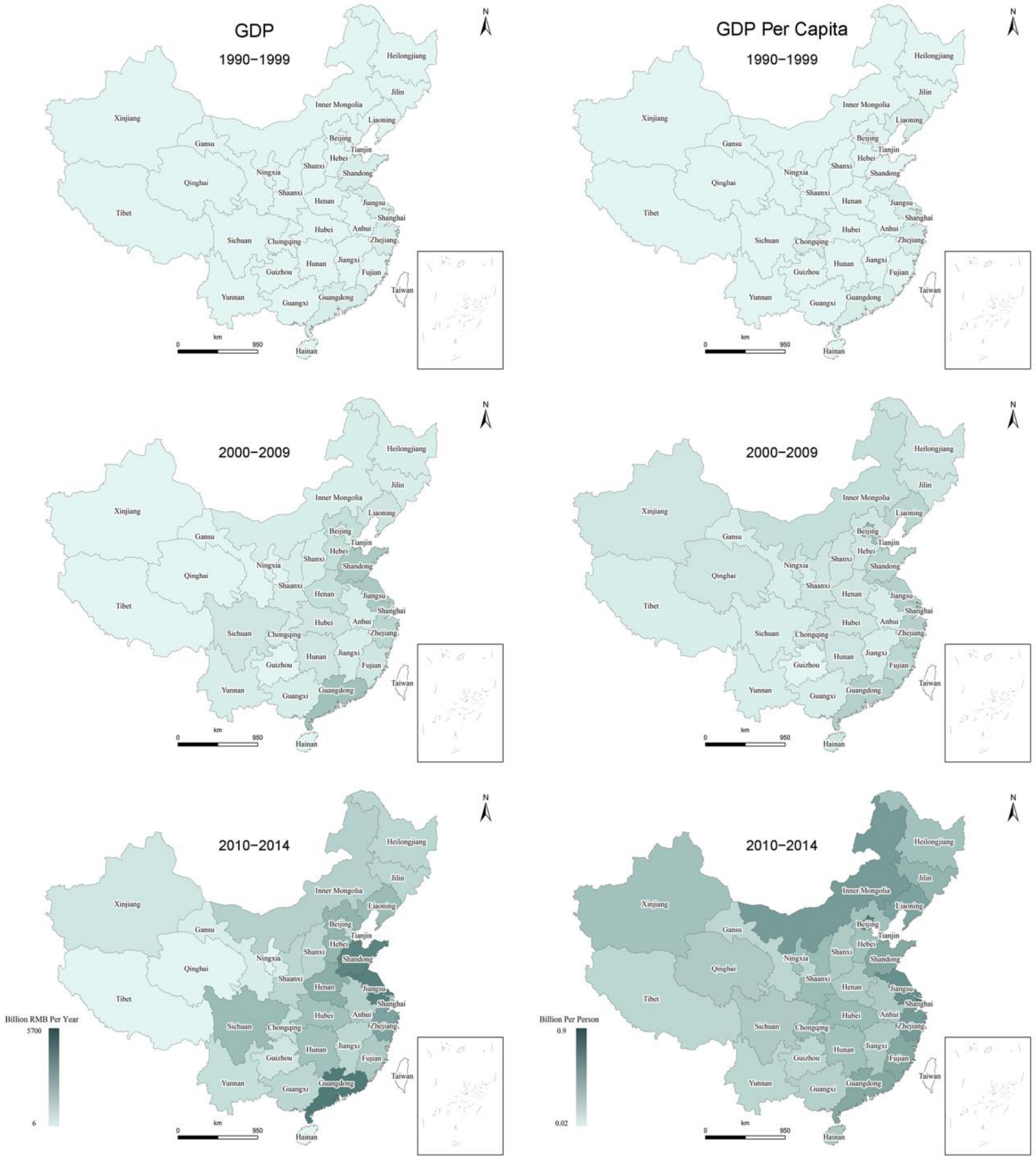


**Technical Appendix Figure 3.** Averaged annual GDP and GDP per capita in each province of China in 1990–1999, 2000–2009 and 2010–2014.

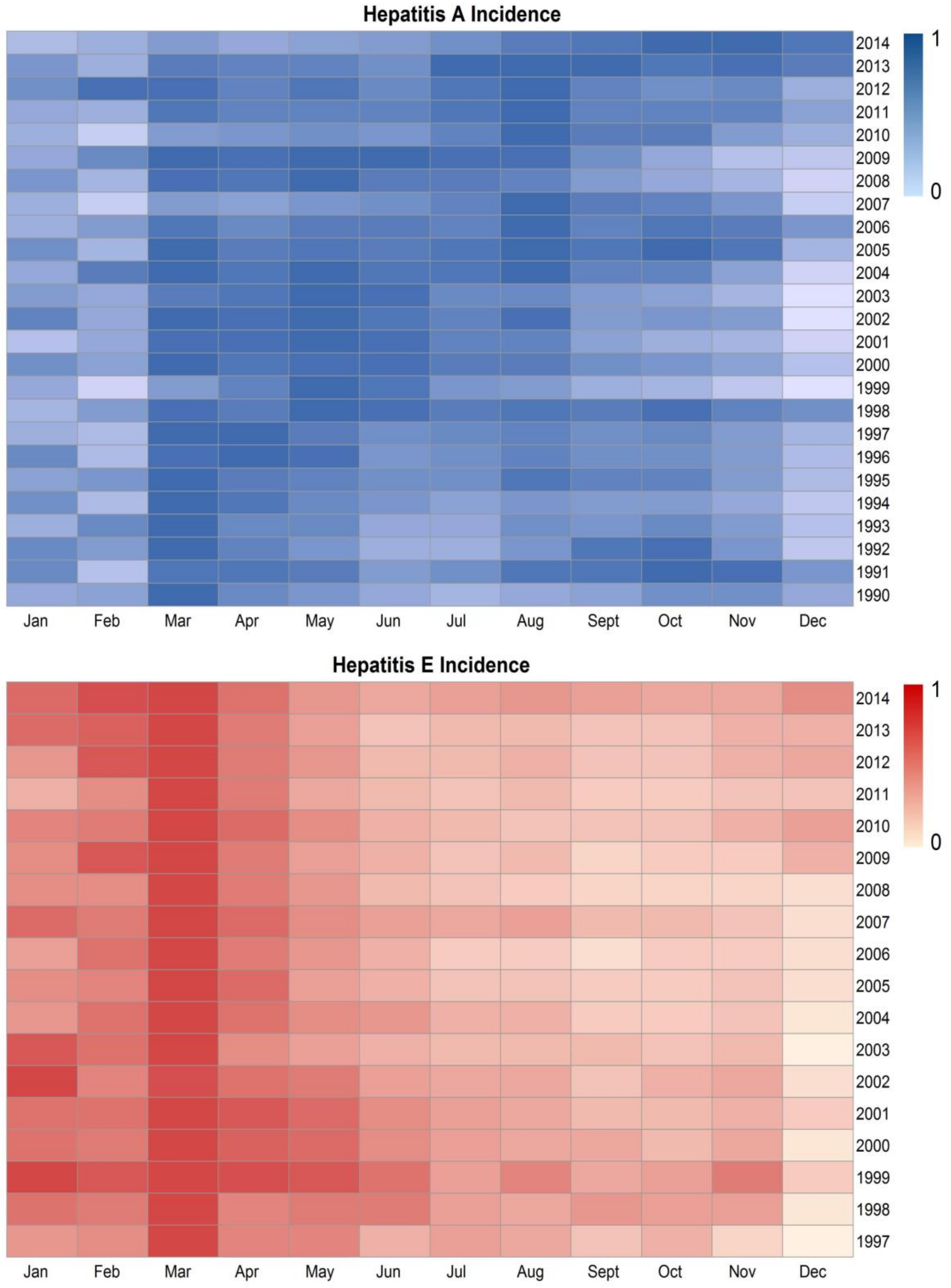


**Technical Appendix Figure 4.** Seasonal patterns in incidence of notified hepatitis A (upper panel) and hepatitis E (lower panel) cases by year in China from 1990 through 2014. The monthly incidence was calculated for each year and then divided by the maximum monthly incidence in each year.

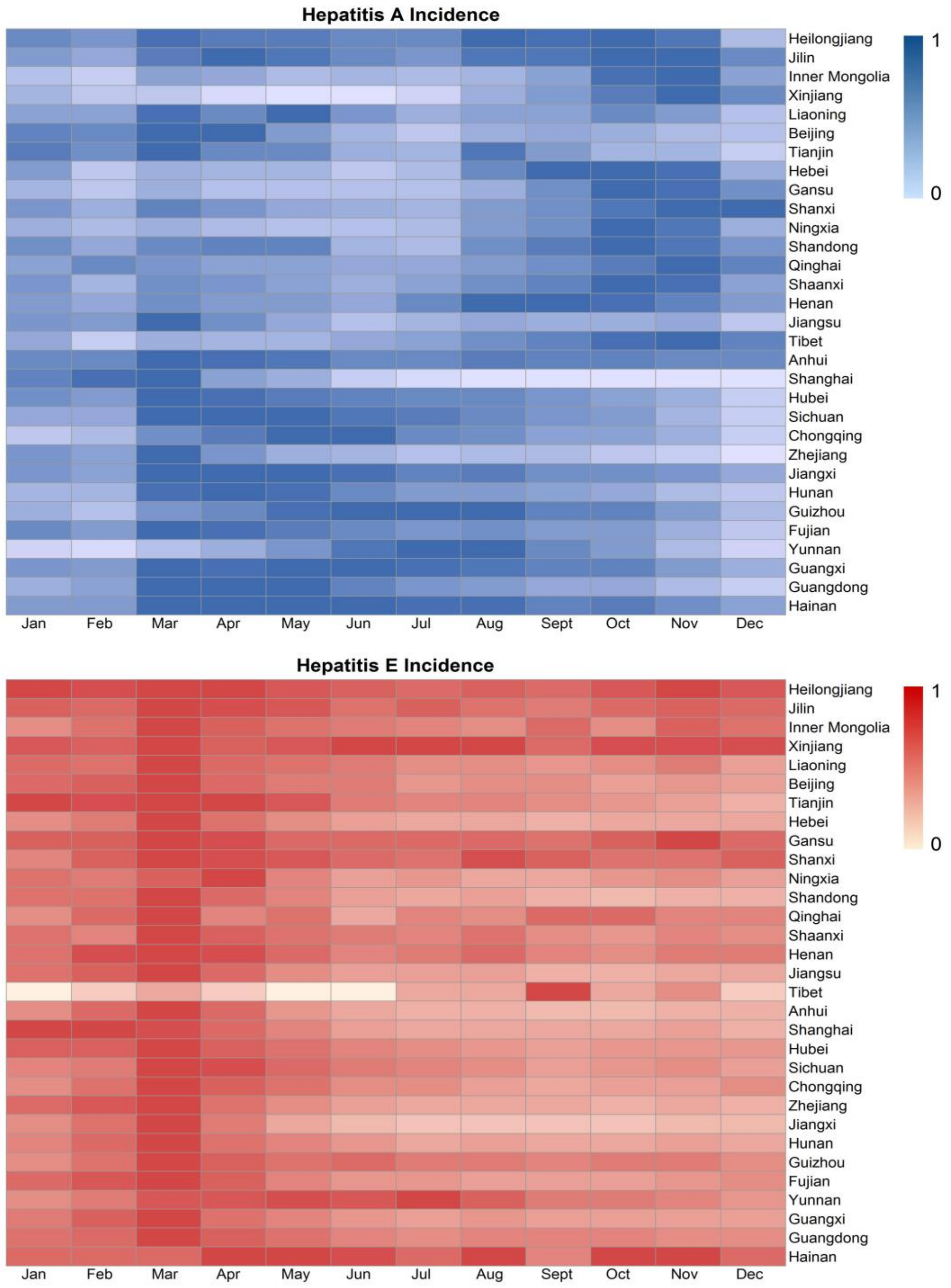


**Technical Appendix Figure 5.** Seasonal patterns in incidence of notified hepatitis A (upper panel) and hepatitis E (lower panel) cases by province from 1990 through 2014. The monthly incidence was calculated for each province and then divided by the maximum monthly incidence in each province. The provinces are sorted by latitude from north to south.
